# Adamant: a JSON schema-based metadata editor for research data management workflows

**DOI:** 10.12688/f1000research.110875.1

**Published:** 2022-04-29

**Authors:** Ihda Chaerony Siffa, Jan Schäfer, Markus M. Becker

**Affiliations:** 1 Leibniz Institute for Plasma Science and Technology (INP), Greifswald, Felix-Hausdorff-Straße 2, 17489, Germany

**Keywords:** Research Data Management, JSON Schema, FAIR Principles

## Abstract

The web tool Adamant has been developed to systematically collect research metadata as early as the conception of the experiment. Adamant enables a continuous, consistent, and transparent research data management (RDM) process, which is a key element of good scientific practice ensuring the path to Findable, Accessible, Interoperable, Reusable (FAIR) research data. It simplifies the creation of on-demand metadata schemas and the collection of metadata according to established or new standards. The approach is based on JavaScript Object Notation (JSON) schema, where any valid schema can be presented as an interactive web-form. Furthermore, Adamant eases the integration of numerous available RDM methods and software tools into the everyday research activities of especially small independent laboratories. A programming interface allows programmatic integration with other software tools such as electronic lab books or repositories. The user interface (UI) of Adamant is designed to be as user friendly as possible. Each UI element is self-explanatory and intuitive to use, which makes it accessible for users that have little to no experience with JSON format and programming in general. Several examples of research data management workflows that can be implemented using Adamant are introduced. Adamant (client-only version) is available from: https://plasma-mds.github.io/adamant.

## Introduction

The demand of funding organizations and publishers to make research data discoverable, accessible, interoperable, and reusable in the sense of the FAIR principles ^1^ and the associated need for digitization and automation of research data management (RDM) processes pose new challenges for some research fields. In particular, for smaller laboratories in physics departments that operate away from large-scale experiments in astrophysics, or high-energy physics, new processes for research data management must be introduced and established. While the aspects of findability and accessibility can be realized without much effort by means of a data publication on a generic platform, the interoperability and reusability of the (domain-specific) data and metadata often pose a major challenge and requires certain conventions and standards in the communities. There are hardly any common research data management processes in areas such as optics and low-temperature plasma physics, and handwritten laboratory notebooks are still the standard in many places. ^2^ On the other hand, electronic laboratory notebook (ELN) systems, repositories, collaborative tools, and (meta-)data standards already exist that can support the implementation of the FAIR principles and Open Science practices. A major challenge is to integrate these tools and standards into the everyday research activities. This is especially challenging when extremely diverse needs have to be addressed at institutions, no specific standard procedures and (meta-)data standards exist, and only a few scientists at a time are dealing with similar instruments and data.

Adamant has been developed to support especially these often small laboratories and departments in the implementation of digital research data management processes and the step-by-step adoption of the FAIR principles. To this end, a tool is provided for the easy use or compilation and creation of domain-specific metadata and metadata schemas based on the widely used JavaScript Object Notation (JSON) schema standard, which recently has been gaining traction in several scientific communities. ^3^^–^^7^ The use of JSON schema in Adamant enables straightforward validation of the metadata ensuring its quality. ^8^^,^^9^ Furthermore, storing the metadata in JSON format maintains the adaptability of the created metadata with different RDM tools and processes, and increases the findability of the research data to which the metadata is attached, in view of the fact that JSON documents are human- and machine-readable. ^8^^,^^10^^,^^11^ An Application Programming Interface (API) based integration with other systems enables the seamless embedding of Adamant into institutional research data management processes. This positions Adamant directly alongside electronic laboratory notebook systems and makes it suitable for ensuring standards-compliant documentation of scientific studies as early as the planning and execution of experiments.

In its current form, Adamant (v1.0.0) offers various features that can support diverse workflows within RDM activities. The features are as follow:

• Rendering of interactive web-form based on a valid JSON schema
• User-friendly editing process of the rendered web-form and the corresponding schema
• Creating a valid JSON schema and web-form from scratch
• Live validation for various field types
• Quick re-use of existing schemas from a list
• Downloadable JSON schema and its form data
• API-based integration as various form submission functionalities

## Methods

This section describes the thought process of selecting the technology stack for the development of Adamant, and the implementation details of the tool’s functionalities. Subsequently, detailed descriptions on how to set up and operate Adamant are described. The code is available from GitHub and is archived with Zenodo. ^29^

### Implementation

**Technology stack and architecture**

Adamant has been developed as a web-based tool. This decision was motivated by the end user experience, where end users can use Adamant directly on a web browser available on their machines and handheld devices with no prior installation and configurations required. We considered several things when choosing the right technology stack for developing Adamant, such as the availability and ease of use of the technology, whether its community is active and big, and the possibility of the technology still being relevant in the coming years, often indicated by its adoption rate and popularity. For these reasons, we adopted a technology stack consisting solely of open-source software, such as ReactJS ^12^ and Flask ^13^ (as the main development frameworks). This stack utilizes two of the most popular programming languages, namely JavaScript and Python ^14^ (Python Programming Language, RRID:SCR_008394). On top of that, we utilized Docker (Docker Desktop, RRID:SCR_016445) and Docker Compose for straightforward packaging and deployment of the developed tool. ^15^ Like a typical web tool or application, Adamant consists of front-end and back-end layers. The front-end part serves as the presentation layer in the form of a Graphical User Interface (GUI) for end users to interact with. The back-end part is a server-side part of the tool with the main task of providing processes that are not suitable to be run on the client-side ^[^ ^1^ ^]^. Lastly, the front-end and back-end layers are bridged using an API built upon the Hypertext Transfer Protocol (HTTP) request methods, such as POST and GET.

The front-end has been developed in the Node.js ^16^ (v14.15.5) JavaScript runtime environment making use of ReactJS (v17.0.2), which is a popular JavaScript library for developing a highly interactive browser-based GUI. ReactJS is combined with the Material-UI library ^17^ (v4.12.3), which contains many pre-existing user interface (UI) components. The pre-existing components can be modified to one’s need straightforwardly thus accelerating the UI development. The main bulk of the features are implemented on the front-end. This includes the automatic rendering process of a JSON schema into a web-form, form editing logic (editing of the rendered web-form or creating one from scratch), and input validation processes. The back-end is written in Python (v3.8.7) using the Flask library (v2.0.2). Flask is a lightweight web framework and straightforward to use making it suitable for the scope of Adamant development. The back-end’s tasks include providing the front-end with available JSON schemas from the server, data preparation, necessary e-mail notification for users, and API calls for communication with external applications (API based integration), e.g., ELN and online data repository systems.

**Figure 1.**
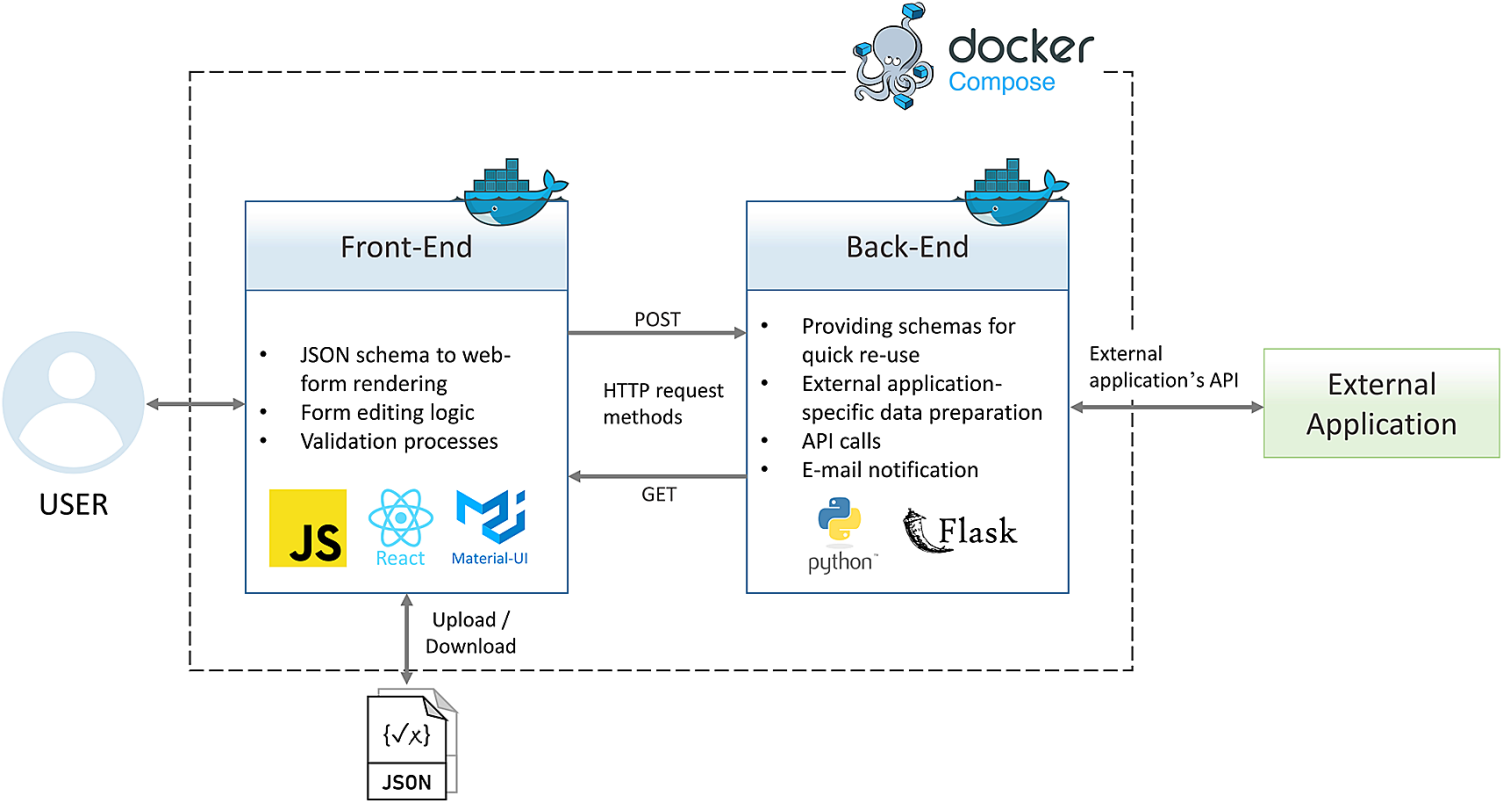
Overview of Adamant’s software architecture. API, Application Programming Interface; JSON, JavaScript Object Notation; HTTP, Hypertext Transfer Protocol.

Figure 1 provides an overview of Adamant’s software architecture and the main functionalities of each application layer along with the corresponding technologies.

**Front-end: JSON schema to editable web-form**

A JSON schema, like every other JSON document, contains key (word)-value pair instances, where the value parts may contain another set of keyword-value pairs, i.e., nested objects. ^8^^,^^10^^,^^11^ In a JSON schema, there are a number of schema-specific keywords that are mainly used for validation and annotation of the elements in a schema. ^8^^,^^9^ For example, a typical set of schema-specific keywords found in a JSON schema and its sub-schema contains $id/id, $schema, title, type, and properties keywords. ^9^ On many occasions, more specific keywords are used to further annotate or describe the elements in a schema. The specific functions of these keywords are dictated by the schema specification version a JSON schema is specified in, which is declared at the very beginning of the schema under the $schema keyword. A comprehensive documentation of the JSON schema standard can be found at https://json-schema.org/. An example of a JSON schema with the specification version draft 4 is shown in Listing 1, which contains the typical keywords stated earlier, and several field elements. One of the main features of Adamant is to create a web-form representation of a given JSON schema, where users can fill it in and create a JSON document containing the form data for anything they want to describe, e.g., a lab experiment or a dataset. It is worth noting that there are several freely-available libraries and tools for JSON form rendering that conform to the JSON schema specifications. ^18^^–^^21^ Unfortunately, the available libraries and tools, though some offer direct editing of the schema, do not provide a user-friendly interface for schema (or form) creation and editing right out of the box as what we have envisioned with Adamant. Therefore, we decided to build a custom JSON schema form renderer from the ground up in order to achieve this, which allows us to maintain full control as well as flexibility during the development of Adamant’s current and future features. At the time of writing this paper, Adamant (v1.0.0) supports the rendering and editing of JSON schemas with a specification version draft 4 or 7.

**Listing 1.**
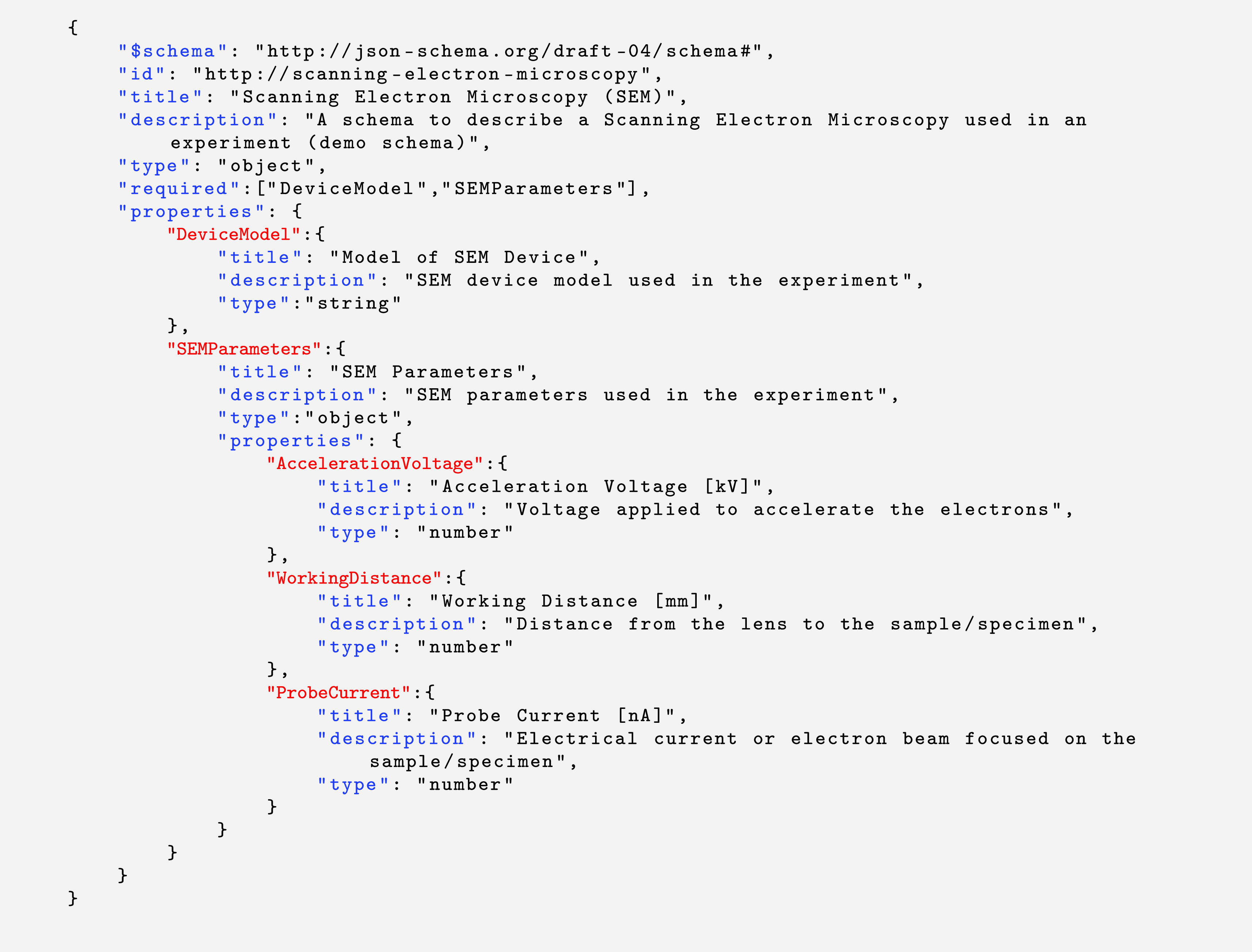
Example of a draft-4 JSON schema containing typical schema-specific keywords presented in blue with their values presented in black, and field element keywords presented in red. JSON, JavaScript Object Notation.

**Figure 2.**
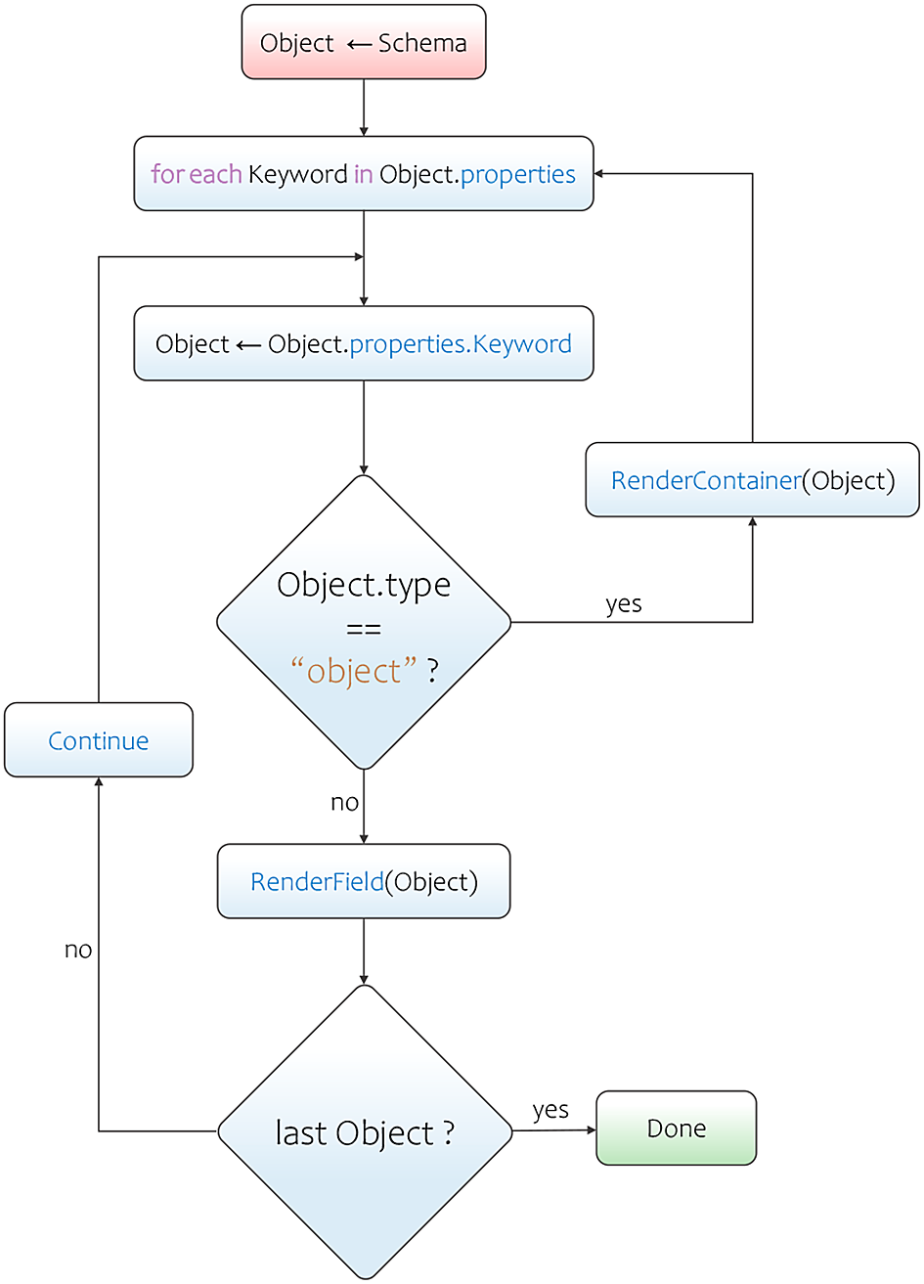
JSON schema to web-form rendering flowchart. JSON, JavaScript Object Notation.

Adamant employs a recursive form field rendering process that makes accessing of the keyword-value pairs located within nested objects possible. The rendering process starts by determining the value of the type keyword in the uppermost object (or parent object) of the schema. The type keyword in this location commonly has a string value of object. This value indicates that a properties keyword is present within the same location. The properties keyword’s value is a set of keyword-value pairs (an object), where each pair contains the information that the process needs to render the field. As the process iterates over these keyword-value pairs, it renders a certain form field type according to its type keyword’s value. The acceptable types in a JSON schema are string, number, integer, boolean, array, and object. If the process stumbles upon a field keyword that has the type of object again, then a container will be rendered, and the recursion process is initiated. This process iterates over the current properties keyword’s value following the same procedure as before, and renders each field within the newly created container. In this way, the process will not terminate until all form fields are rendered. This is a condition where the process does not find any field keyword with the type of object anymore. Apart from the type keyword’s values, the renderer also makes use of other keywords’ values to adorn the form field with useful information, such as the values of title and description keywords (annotation keywords). The main steps of this form field rendering process is also represented in a flowchart diagram as shown in Figure 2.

Another main feature of Adamant is the capability of editing the rendered fields. This feature allows users to change the values of relevant keywords in the schema, and re-order the rendered fields within the same container. To achieve this feature, apart from the rendering of the form fields, each rendered field is also supplied with several editing interfaces along with the editing and input handling logic. Creating a schema (or form) from scratch is another feature made possible by using the same principle of this form editing feature, which basically presents the user with a blank schema and lets them add the field elements as required.

For a certain use case, a file upload functionality can be included in a rendered form. This functionality is intended as an alternative way of enriching the metadata when texts are no longer sufficient. For example, an experiment may have a graphical description of its set-up. In this case, the user can include an image file within the metadata using this file upload functionality. To add this functionality, a string-type field element, with the addition of “contentEncoding”:“base64” keyword-value pair, must be specified in the schema. This particular keyword-value pair will inform the rendering function to create a file upload field element, where the selected file is read as a string of base64-encoded binary data. Fortunately, adding the file upload functionality can be done in only a couple of mouse clicks in Adamant. For a smooth experience, the uploaded file is recommended to have the size of less than 500 kilobytes.

Lastly, Table 1 presents a complete list of the implemented field types and their relevant keywords in the current version of Adamant (v1.0.0).

**Table 1.** Implemented JSON schema field types and their relevant keywords in Adamant v1.0.0. Note that the id keyword only works with the JSON schema specification version draft 4, whereas $id is used for the newer specification drafts. Lastly, the contentEncoding keyword is intended to be used with the specification version draft 7 or newer. JSON, JavaScript Object Notation.

Field type	Implemented keywords	Note
String	title, id, $id, description, type, enum, contentEncoding, default, minLength, maxLength	contentEncoding can only receive a string value of “base64”
Number	title, id, $id, description, type, enum, default, minimum, maximum	
Integer	title, id, $id, description, type, enum, default, minimum, maximum	
Boolean	title, id, $id, description, type, default	
Array	title, id, $id, description, type, default, items, minItems, maxItems, uniqueItems	
Object	title, id, $id, description, type, properties, required	

**Front-end: JSON form data validation using Ajv**

As the user fills in the form, a JSON document containing the entered data is created and updated accordingly and simultaneously, this JSON document is called JSON form data. Upon finishing the form filling process, the created JSON form data can be used for other operations. To ensure the correctness or quality of the entered data, the JSON form data is validated against its schema prior further operation. For this validation purpose, Adamant takes the advantage of the Ajv library (v8.8.2). ^22^ By using this library, any discrepancies between the JSON form data and its schema can be identified. For example, if a field is required to be filled and the user does not provide any input, then the process will throw a validation error with relevant error messages. Many other keyword specific validations are included in this library, which helps with ensuring the quality of the form data with respect to its schema.

**Back-end: API-based integration**

Many existing web applications provide APIs for seamless integration with other (external) web tools or applications. For instance, web applications that are commonly used in the RDM community include online data repository systems, ELNs, collaborative and version control applications, etc. These applications, more often than not, provide API endpoints that are straightforward to use. Several web tools have also been developed each addressing certain challenges in RDM, such as COPO, ^3^ and Dendro. ^23^ Adamant is equipped with a back-end layer whose main purpose is to ensure the possibility of seamless integration with such web applications. The back-end is written in Python (v3.8.7) using the Flask library (v2.0.2), which is advantageous given numerous web applications provide Python libraries for their API calls. The connection between Adamant’s front-end and back-end is achieved primarily using the POST and GET HTTP request methods. The POST method is used to send data from the front-end to the back-end, which is then pre-processed (if needed) and relayed to the external application (using the provided API). The GET method is used when the client-side requires information or data only available in the server-side or from an external application, e.g., when retrieving schemas from the server, or retrieving tags and database items from an external application. With this straightforward approach, various functionalities can be implemented in the server-side that can help with realizing application-specific RDM workflows.

### Operation

Many features of Adamant can be explored directly in the client-only version available online at https://plasma-mds.github.io/adamant. This version only lacks the submission-related features, which require the server-side to operate. To access the full features, Adamant can be set up and deployed on a local machine or a server that allows for running both the client and server sides. In most instances, Adamant is not resource intensive, and can be used on a typical smartphone and laptop. However, for deployment and development, we recommend to set up Adamant on a system with at least a 64-bit CPU (with 4 cores) and 8GB of RAM. At the time of writing, Adamant has been tested on Firefox (91.5.1esr), Chrome (v97.0.4692.99), and Chrome Mobile (v97.0.4692.98) web browsers. The following subsections introduce the deployment process of Adamant and how users can interact with it.

**Setting up Adamant on a local machine**

For development purpose, Adamant can be set up on a local machine. For a Linux system (tested on Ubuntu 20.04.4 LTS), the following steps can be taken for this:

1. $ git clone https://github.com/plasma-mds/adamant.git — clone the repository
2. $ cd adamant — go to adamant project directory
3. adamant$ npm install — install the dependencies for the client-side
4. adamant$ cd backend — go to backend directory
5. adamant/backend$ python3 -m venv venv — create a python virtual environment
6. adamant/backend$ source./venv/bin/activate — activate the virtual environment
7. adamant/backend$ pip install -r requirements.txt — install the dependencies for the back-end
8. adamant/backend$ ./venv/bin/flask run --no-debugger — start the back-end
9. Start a new terminal
10. adamant$ npm start — on the new terminal, in the adamant project directory, start the client-side

Typically, a web-browser presenting the client-side will open automatically following the last command. In any case, Adamant can be accessed at http://localhost:3000.

**Deploying Adamant using Docker**

Adamant is intended to be deployed on an institutional server in the internal network. In this way, anyone who is connected to the institute’s network can use Adamant directly. We recommend using Docker to deploy the production build of Adamant, which can be done with the following steps:

1. $ git clone https://github.com/plasma-mds/adamant.git — clone the repository
2. $ cd adamant — go to adamant project directory
3. adamant$ docker − compose build — build the docker images for both back-end and front-end
4. adamant$ docker − compose up -d — start both client and server containers, i.e., the whole system

By default, the deployed system can be accessed at http://localhost:3000.

**General overview of the Adamant UI**

**Figure 3.**
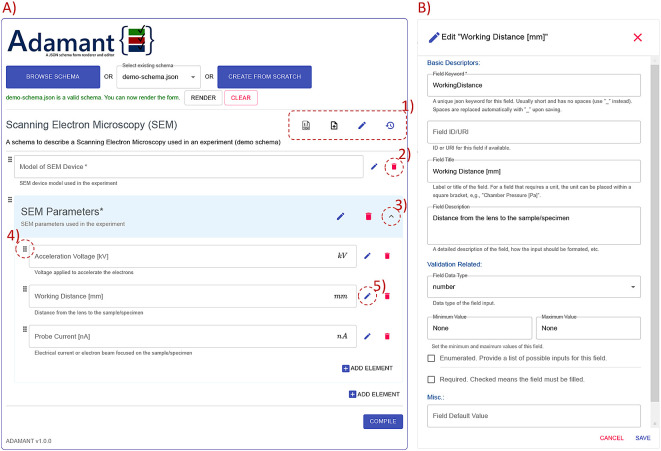
Overview of the Adamant UI with a rendered web-form based on the schema in Listing 1 as an example. (A) Main corpus of the UI; (1) from left to right: JSON schema viewer, auto-populate form, edit schema description, revert all changes; (2) remove form field; (3) collapse or expand the field container; (4) field drag handle; (5) edit field description and (B) field editing panel (as a pop-up on top of the main UI) triggered by clicking (5) the edit button. UI, user interface; JSON, JavaScript Object Notation.

The general overview of the Adamant UI is presented in Figure 3. Adamant renders a given JSON schema into a web-form by uploading the schema from a local drive using the “BROWSE SCHEMA” button, or if already existing, the schema can be selected from a list next to the browse button. Creating a JSON schema from scratch is possible by clicking the “CREATE FROM SCRATCH” button. After uploading or selecting the schema, the schema is checked by Adamant to see whether the schema file type and structure are valid. If the schema is valid, the schema can be rendered by clicking the “RENDER” button. The rendering can be undone by clicking the “CLEAR” button, which also discards the current schema.

As a demonstration, Figure 3 shows the rendered web-form based on the schema represented in Listing 1. Each rendered field is shown along with its editing interfaces, and the field container’s header is rendered in blue for a quick identification. Furthermore, the field container can be expanded and collapsed for a good user experience. The editing interfaces found in each field consists of edit and remove button icons, and a drag handle icon. These interfaces allow the user to change the values of relevant keywords in the schema (directly affecting the rendered field), and re-order the rendered fields by dragging and dropping the field to the desired order within the same container. The changes are reflected to the rendered form immediately, which promotes a What You See Is What You Get (WYSIWYG) application experience for the users. On top of that, each field container is equipped with the “ADD ELEMENT” button for adding a new field within the corresponding container. By default, Adamant renders the form in the edit mode right after a schema is rendered. The editing mode can be concluded by pressing the “COMPILE” button, which removes all editing interfaces (thus also removes the editing functionalities) and readies the form for use. In case of needing to edit the form again, the user is able to initiate the editing mode again, and without losing the already entered data.

**Adding and removing schemas**

A number of schemas can be fixed in the back-end. The back-end serve these stored schemas to the front-end, from which the end users can select. This is important for a quick re-use of the schemas, especially the ones that are regularly used. For this purpose, one can simply add the desired schemas to adamant/backend/schemas/ directory. Likewise, a schema can be removed from this directory. The list of the schemas in the front-end is updated every time the front-end is refreshed.

## Use cases

At its core, Adamant is a JSON schema form renderer-editor and JSON form data creator presented in an interactive and user-friendly UI. It can be operated by anyone with no prior knowledge of JSON format and coding. This section elaborates the general and *ad hoc* use cases of Adamant that it can be suited to.

### Collection of structured and standardized metadata

The creation or use of a valid JSON schema and the collection of corresponding form data being consistent with the schema can be considered as the general use case of Adamant, which simplifies the collection of structured and standardized metadata. This is particularly relevant for laboratories that are not embedded in large research clusters providing access to established standards and digital research data management workflows. On the one hand, the ability to define required metadata fields and formats supports metadata quality assurance, and on the other hand, storing metadata in JSON format enables further (automated) processing of metadata. This both directly contributes to the “FAIRness” of metadata.

### Creation of an eLabFTW experiment with schema-compliant metadata

The use case introduced here aims to highlight how the previously described ability to interface with external systems through API-based integration can be used to generate schema-compliant experiment descriptions in eLabFTW. The open source ELN system eLabFTW ^24^^,^^25^ (elabFTW, RRID:SCR_013971) is becoming increasingly popular, especially at universities and research institutions. Given Adamant’s features (JSON schema-based metadata creation, input validations, etc.), one can see why it could be beneficial to collect structured metadata using Adamant rather than doing it directly in the ELN system, especially when the ELN system is designed to be as general as possible in order to accommodate various kinds of experiments from different scientific fields. Note that eLabFTW already offers the possibility to use predefined templates and to edit JSON files. However, a user-friendly possibility to work directly with JSON schemas and to validate the entered metadata on the basis of these predefined schemas is missing so far.

**Figure 4.**
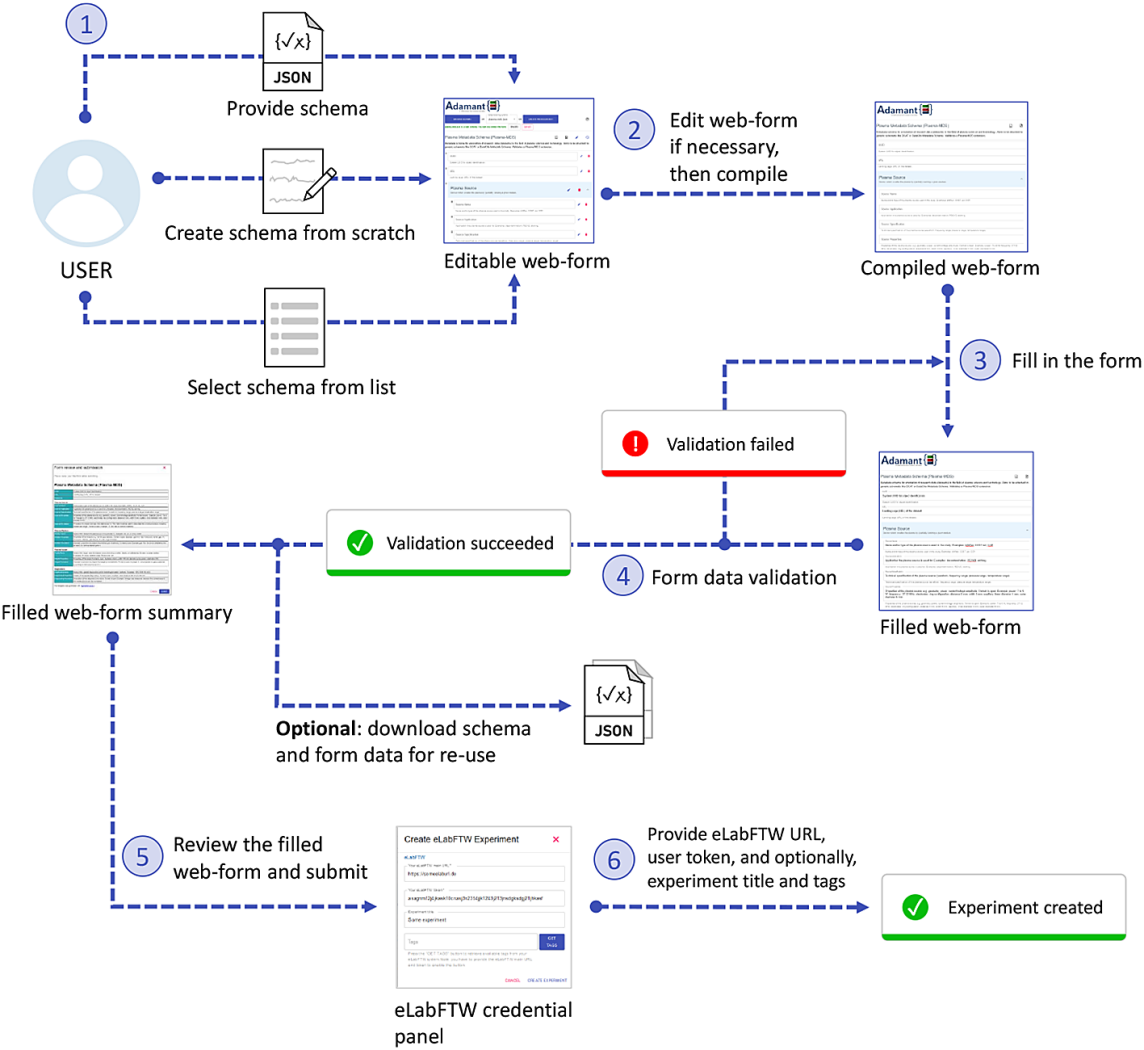
Workflow for creating a new experiment in the eLabFTW system with Adamant.

Figure 4 shows the workflow for creating an experiment using Adamant in the eLabFTW system from the end-user’s perspective. The user generates the experiment form by uploading their experiment schema, or selecting it from the list if it already exists. Alternatively, the user can create the form from scratch. If necessary, the user can edit the form (affecting the given schema). For example, changing the title of a certain field, removing a field, etc. When satisfied, the user can compile the rendered form and proceed to fill it in. Upon completing the form, Adamant validates the entered data against the given schema and notifies the user if discrepancies between the entered data and the schema are found, which the user can rectify thereafter. When the entered data are valid, the user can proceed to review the form. After a thorough review, the user can proceed and submit the form. During the submission process, the user will be prompted to input the URL of their eLabFTW system and their eLabFTW API token. Optionally, the user can provide the title and the tags for their experiment. Upon submission, Adamant’s back-end prepares the content and creates a new experiment in eLabFTW accordingly, making use of the available eLabFTW API for python, namely elabapy (v0.8.2). ^26^^,^^27^ The user will be notified upon a successful submission, and the created experiment can be viewed in the eLabFTW system.

**Figure 5.**
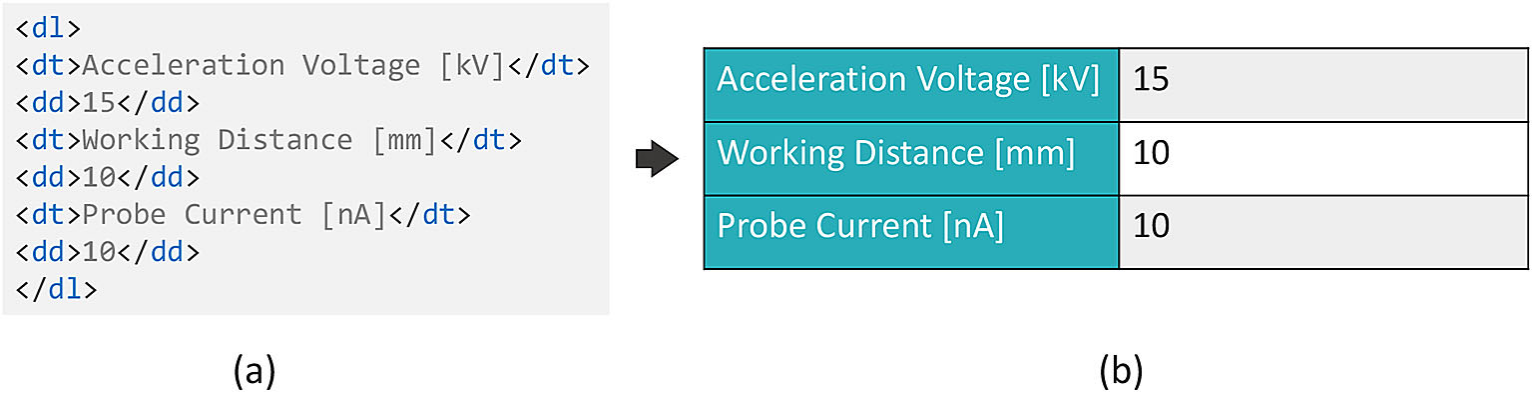
HTML description list rendering in eLabFTW. (a) Title-value pairs in the HTML description list format, and (b) the rendered description list with a custom CSS styling. CSS, Cascading Style Sheets.

The eLabFTW system (v4.2.2) makes use of the TinyMCE text editor ^28^ for free text descriptions of experiments. Here, HTML content can be loaded and rendered. Based on this functionality, the present implementation of the described workflow generates an HTML description list content based on the submitted JSON form data and its schema. The title of the field (obtained from the schema) is used and paired with the value of this field, as shown in Figure 5(a), where the titles are encapsulated within the <dt> tags, the values within the <dd> tags, and the complete pairs in the <dl> tags. The description list is rendered in a way to attain or simulate the look of a form, as presented in Figure 5(b), which increases the readability of the content. This result is achieved by using a custom Cascading Style Sheets (CSS) styling, which can be provided to the eLabFTW system. The generation of this description list content is carried out on Adamant’s front-end side. Note that it is equally possible to upload the experiment information prepared in Adamant directly to the eLabFTW experiment in JSON format. However, this would present it in the form of additional information to the experiment description. Since we consider the structured metadata to be the main component of the experiment documentation, the described workaround based on the HTML description lists in the main body has been chosen. Still, the schema, JSON form data, and uploaded files (if available) are sent as attachments to the eLabFTW experiment. In this way, the schema and form data that describe the experiment are preserved and available for re-use, while retaining the high readability of the experiment body for the user.

### Submission of metadata for research lab workflow

Research institutions are often equipped with advanced laboratory instruments, such as Scanning Electron Microscopy (SEM) and Mass Spectrometry (MS) instruments just to name a few. These instruments are pivotal for various research investigations in many scientific fields. However, not every researcher at the institute is able to operate and has access to such instruments. To this end, we propose a job request workflow using Adamant that also incorporates the use of eLabFTW for experiment documentation.

**Figure 6.**
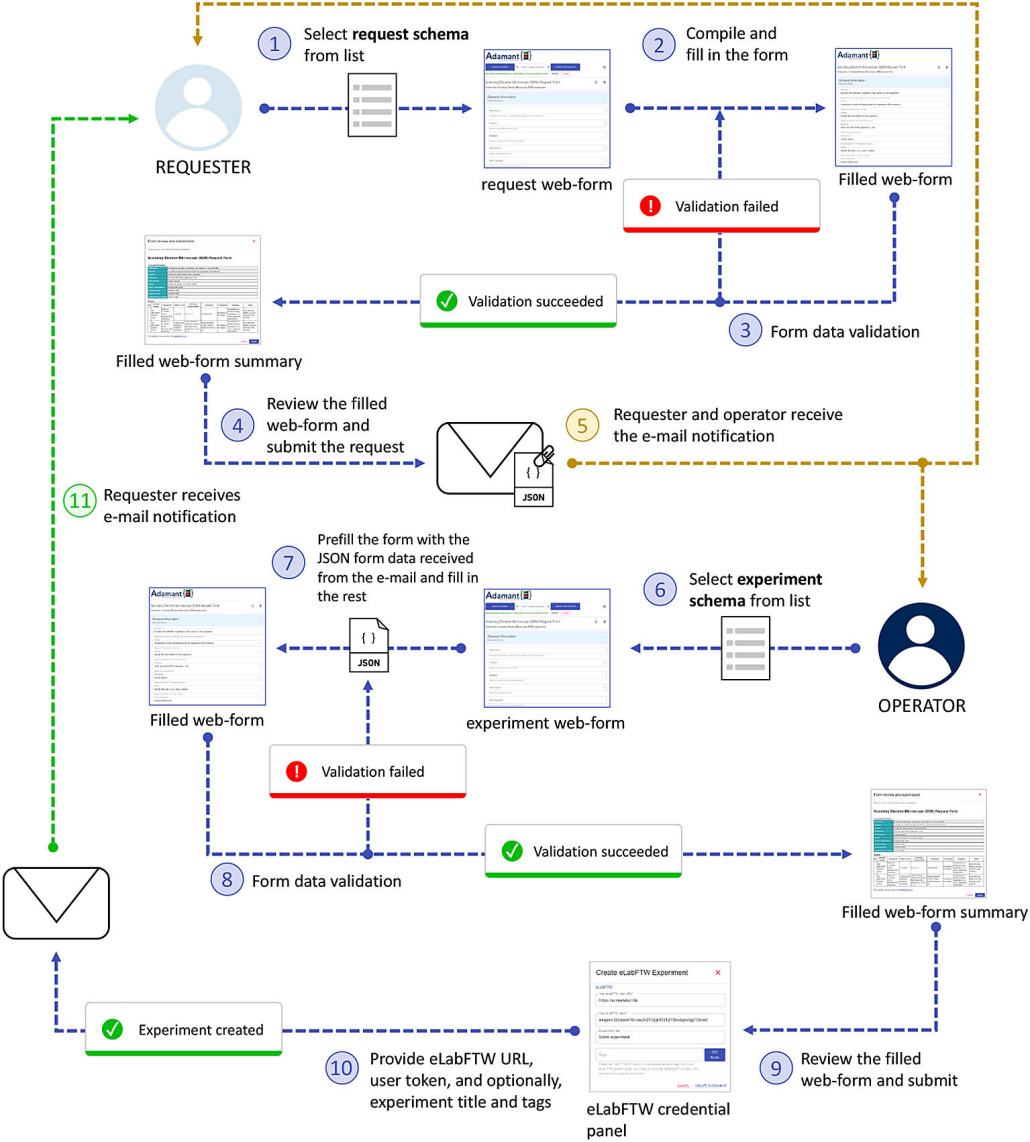
Job request workflow using Adamant involving two users: a requester and an operator. JSON, JavaScript Object Notation.

The job request workflow represented in Figure 6 aims to streamline the process of requesting a job or an investigation of an object of interest using a laboratory instrument that needs to be carried out by the designated instrument operator. From Adamant’s perspective, this workflow involves two users, namely the researcher who wants to get something done with an instrument they do not know how to use (or do not have access to), denoted as the “requester”, and the instrument operator, denoted as the “operator”. From the documentary point of view, the requester provides the information required to execute the job, while the operator can add metadata related to the instrument and the analysis method. Hence, the schemas used for this workflow can be divided into two, the request schema and the experiment schema. The request schema is used by the requester, and is a subset of the experiment schema including only the form fields for the request details. The experiment schema is used by the operator, which contains every form field necessary to describe the experiment using the selected instrument. Examples of a request schema and its corresponding experiment schema (complete schema) are available here and here, respectively. Both users are notified automatically per e-mail for relevant events within the workflow, such as when the requester submits the job request and the operator starts working on the request. The e-mail notifications are made sure to work accordingly by including the necessary information of the requester and operator within the used schemas, and by setting up the e-mail notification configuration for the relevant schemas. An example of the e-mail notification configuration file can be found in adamant/backend/conf/, in which each configuration parameter is explained. For using more than one job request workflow, one can simply add another schema-specific configuration into the confList keyword. The right configuration is then automatically selected based on the relevant schema titles.

## Conclusions

We have developed a web-based tool named Adamant with the aim of easing the implementation of digital research data management processes. With this, we intent to support the adoption of the FAIR data principles in independent labs, which do not (yet) have access to established research data management infrastructures and domain-specific standards. Adamant provides straightforward compilation and creation of metadata and metadata schemas based on the JSON schema standard, and API-based integration with other available research data management software tools. The intuitive and self-explanatory UI of Adamant increases the accessibility for users with little to no experience with JSON format and programming in general. A flagship implementation of Adamant is the use of the tool, generally, in a core facility or laboratory of a research institute that often hosts advanced scientific equipment, such as the scanning electron microscopy instrument. This is often an advanced expertise that different teams want to use, while a small competence group (experts in such an instrument) ensures that the investigation tasks are optimally processed. Such a use case is demonstrated in the Adamant’s submission of metadata for research lab workflow. With that as an example, Adamant can be suited to many specific use cases by extending it with relevant submission functionalities.

## Data availability

### Underlying data

All data underlying the results are available as part of the article and no additional source data are required.

## Software availability

• Adamant (client-only version) available from: https://plasma-mds.github.io/adamant
• Source code available from: https://github.com/plasma-mds/adamant
• Archived source code at time of publication: https://doi.org/10.5281/zenodo.6396182 ^29^
• License: MIT

## Footnotes

^1^
In this paper, the terms “front-end” and “client-side” are used interchangeably, as well as “back-end” and “server-side”.
