# Supplementary material for: Adamant: a JSON schema-based metadata editor for research data management workflows
Source: F1000Res. 2022 Jul 19;11:475. Originally published 2022 Apr 29. [Version 2] doi: 10.12688/f1000research.110875.2 (PMC9178528; doi:10.12688/f1000research.110875.2)
Supplement: Peer review history

# Peer review: Reviewer response for version 2

**DOI:** 10.5256/f1000research.136205.r136390

**Published:** 2022-08-05

**Authors:** Martin Thomas Horsch

**Affiliations:** 1 Norwegian University of Life Sciences, Ås, Norway

Adamant is a tool for editing JSON schemas and creating a data ingest front end from a given JSON schema. Such tools are useful to have, and it is good that the developers are contributing a concise presentation of their work to F1000Research. It is also good to see a demonstration of interoperability with the eLabFTW ELN.

 The issue of its application to graph-based data, which was raised by the previous reviewers as well, will be of interest to readers and users, and here the appropriate mechanism would probably be through JSON-LD. Any support that can be provided in this field would be helpful to many since ontologies etc. are easily written, but it is then the data ingest through web forms, etc. that become the main challenge in making them useful in practice.

 Fig. 2 looks confusing to me; pseudocode convention has it that "<-" normally means "is assigned to," but in the boxes from this figure, such as "Object <- Schema" or "Object <- Object.properties.keyword" it must have a different meaning. I have the suspicion that Fig. 2 must mean something very simple that is just obscured by this visualization.

 The material included under "setting up Adamant on a local machine" and "deploying Adamant using docker" would really best belong into a README file rather than a paper presenting the software - no doubt, however, it will be helpful to users.

Are the conclusions about the tool and its performance adequately supported by the findings presented in the article?

Yes

Is the rationale for developing the new software tool clearly explained?

Yes

Is the description of the software tool technically sound?

Yes

Are sufficient details of the code, methods and analysis (if applicable) provided to allow replication of the software development and its use by others?

Yes

Is sufficient information provided to allow interpretation of the expected output datasets and any results generated using the tool?

Yes

Reviewer Expertise:

Applied ontology, data management, molecular simulation, process data technology, thermodynamics

I confirm that I have read this submission and believe that I have an appropriate level of expertise to confirm that it is of an acceptable scientific standard.

# Peer review: Reviewer response for version 1

**DOI:** 10.5256/f1000research.122529.r136576

**Published:** 2022-06-07

**Authors:** Frank Krüger

**Affiliations:** 1 Institute of Communications Engineering, University of Rostock, Rostock, Germany

The FAIR Guiding Principles require, among other things, the provision of rich metadata for research data and the generating processes. For this purpose, structured information that reuses existing vocabulary in a standardized way is desired. While such thorough documentation improves the reusability of the research data, it demands knowledge about appropriate vocabularies (e.g. DCAT, DDI...) and some minimum technical understanding of structured documentation (e.g. JSON, JSON-LD, RDF...) of researchers from all scientific domains. Furthermore, mechanisms to ensure the provision of some minimal required information are necessary. These requirements increase the complexity of providing FAIR data and create the need for appropriate tool support.

 Adamant is a web-based tool for the provision of structured metadata which guides the researcher through the documentation process and provides a means for validation of the content and completeness by providing a schema-based form. The underlying schema can either be loaded from a library or particularly designed for the research process at hand. By providing a rich API, Adamant satisfies further requirements for the integration into complex workflows based on virtual research environments.

 From the technical perspective, Adamant employs JSON Schema, a technology to specify the structure of JSON documents including required and optional information. A web-form, based on Python/Flask, is generated from the schema that is presented to the end-user to provide the necessary information. Adamant is publicly hosted for testing purposes and can easily be deployed by using the provided docker image. All code is available from github and archived at Zenodo. The released version worked without any problems for me. I tested the web-form and the integration with elabFTW.

 While I like Adamant and the article very much, I have a few suggestions/questions:

1. Adamant is based on JSON Schema and JSON, but recently I recognized an increase in RDF-based (meta)data management. I was wondering if RDF/S and SHACL would also be a viable base for Adamant and why the authors didn't employ those.
2. I further agree with Felix Shaw (Reviewer 1) about the integration of ontologies for the structured and semantic documentation of research data.

Are the conclusions about the tool and its performance adequately supported by the findings presented in the article?

Yes

Is the rationale for developing the new software tool clearly explained?

Yes

Is the description of the software tool technically sound?

Yes

Are sufficient details of the code, methods and analysis (if applicable) provided to allow replication of the software development and its use by others?

Yes

Is sufficient information provided to allow interpretation of the expected output datasets and any results generated using the tool?

Yes

Reviewer Expertise:

Data Science, Provenance, Metadata

I confirm that I have read this submission and believe that I have an appropriate level of expertise to confirm that it is of an acceptable scientific standard.

# Response

**Published:** 2022-07-12

**Authors:** Ihda Chaerony Siffa

**Affiliations:** Leibniz-Institut für Plasmaforschung und Technologie e.V., Germany

We thank Frank Krüger for their valuable comments. The following are our responses to each individual comment:

1. **Response for comment 1**: From our understanding, it is feasible to use RDF(S) formats and SHACL for generating web-forms and validation. Our arguments on using JSON and JSON Schema instead of RDF(S) and SHACL are (in no particular order):
  - RDF(S)/SHACL are ontology-related standards that are surely the means of choice in cases where well-defined ontologies already exist. Note that Adamant specifically targets use cases for which ontologies or metadata standards do not yet exist.
  - JSON Schema supports a set of data types that are analogous to those found in many programming languages (e.g., Python, JavaScript). This allows straightforward implementation of specific input types and their validations.
  - To the best of our knowledge, the JSON format is the de-facto standard of API data interchange. Since Adamant can be used with different external applications by means of API integration, describing the metadata and its schema in the JSON format seems sensible.
  - Many existing metadata schemas are available in the JSON format.
  - On top of that, we plan on using ontologies, serialized in an RDF format, to describe the schema elements that are used in JSON schemas. A Uniform Resource Identifier (URI) can be assigned to each schema element in the JSON format, which allows the interoperability of these schema elements across both formats.
2. **Response for comment 2**: Please see the response under Reviewer 1’s comments.

# Peer review: Reviewer response for version 1

**DOI:** 10.5256/f1000research.122529.r136392

**Published:** 2022-05-03

**Authors:** Felix Shaw

**Affiliations:** 1 Data Infrastructure and Algorithms, Earlham Institute, Norwich, UK

Standards for experimental metadata are in most cases sub-optimal or non-existent. Being able to describe protocols, samples, instruments, etc. in a consistent way is vital to the reusability of data and the reproducibility of work. This situation will only continue with the prevalence of machine learning techniques in science which rely heavily on labeled data. Adamant is a web tool for authoring generic experimental assays and then filling out such assays. Users are presented with an interface in which they can add fields of different data types, and various input restrictions (e.g. enumerations, character lengths, etc.). The system then creates a JSON document that records these fields and can be used later for validation and display. Users can then fill out these generated forms with real assay data which is validated against the JSON document, and in turn, produces another JSON document with the assay data itself.

 This is a good tool, is well presented, and is easy to use. In functionality, it seems comparable with the ISATools suite of software, although less mature and (arguably) more intuitive. It uses industry-standard software (Python, Flask, React, etc.) and is under an MIT license on Github, meaning anyone can download and modify it. This means if uptake is good, there is a fair possibility of community maintenance. There are precious few tools for authoring metadata schemas, and this is a welcome step in the right direction. I downloaded and tested the docker version and it worked without any problem. I didn't download and run the development version.

 There are some things I would like to see:

- Creating metadata standards is in my experience very much a community effort. Whilst this tool makes the "process" of making metadata standards easy, there needs to be an emphasis on community agreed standards. Otherwise, we risk the proliferation of a multitude of standards that fit only very niche applications.
- Talking of community agreed standards, it would be nice to have a large selection of existing standard schemas available for users to select, and then possibly edit. This would be a big time-saver for a lot of researchers.
- There doesn't seem to be a way of using ontology terms for field names, values, or units. Ontologies are the best way of disambiguating metadata, and services such as EMBL/EBI's Ontology Lookup Service make real-time searching for ontology terms easy. I would like to see this implemented in the authoring stage.
- I have a feeling this will not scale particularly well. It's fine to fill out a web form for a few samples or assays, but in practice, researchers will have hundreds or thousands of records. Filling out web forms on this scale is impracticable. I would like to know the author's views on how to overcome this issue.
- Whilst the authors give the use case of submitting an assay to eLabFTW, it would be nice to see some other integrations, such as with Dataverse, Dspace, or CKAN repositories, which are often used by smaller research facilities (the apparent target audience for this work).

Are the conclusions about the tool and its performance adequately supported by the findings presented in the article?

Yes

Is the rationale for developing the new software tool clearly explained?

Yes

Is the description of the software tool technically sound?

Yes

Are sufficient details of the code, methods and analysis (if applicable) provided to allow replication of the software development and its use by others?

Yes

Is sufficient information provided to allow interpretation of the expected output datasets and any results generated using the tool?

Yes

Reviewer Expertise:

Data Science, Open Science, Metadata Standards, Web Development

I confirm that I have read this submission and believe that I have an appropriate level of expertise to confirm that it is of an acceptable scientific standard.

# Response

**Published:** 2022-07-12

**Authors:** Ihda Chaerony Siffa

**Affiliations:** Leibniz-Institut für Plasmaforschung und Technologie e.V., Germany

We thank Felix Shaw for their insightful and constructive comments and helpful suggestions. Some of the comments are in line with what we already have in mind for further development of the tool which we will include in the next version of the manuscript as an outlook. Nevertheless, we will still attempt to address each individual comment briefly as follows:

1. **Response for comment 1:** We definitely agree and have experienced this matter first hand. At the very early stage, Adamant is intended to be used to create and design the initial experiment schema, which later can be shared/made public in a publicly available collaborative version control platform (like GitHub). Once the schema is made public, the community can participate in the further development and maintenance of the said schema.
2. **Response for comment 2: **Thank you very much for this suggestion. Besides the public maintenance of already available schemas for the domain of plasma technology (see our community effort at https://www.plasma-mds.org ) it is indeed an interesting idea to provide easy access to established standards like Dublin Core, DataCite, etc., via Adamant. We will think further in that direction.
3. **Response for comment 3:** We agree with the comments regarding ontologies and we have already thought of something in this direction. We will definitely implement features related to the use of ontologies in the future release of Adamant. One thing that we have planned is to have a domain-specific ontology that can be attached to the tool where it can be used to populate the domain-specific knowledge graph.
4. **Response for comment 4: **This is true. In the field of low-temperature plasma science and technology, there is rarely any case that requires the researcher to fill out hundreds or even thousands of entries in an experiment. However, to make sure that Adamant can be adopted in different research fields, this requirement is, of course, crucial. One thing that we have in mind is to have a feature of uploading a tabular file, which is then translated to its JSON representation. Such a feature would also enable the collection and further processing of standard-conform metadata in “offline” field experiments.
5. **Response for comment 5: **Currently, we are looking into implementing a feature of dataset publication in the DKAN-based data platform INPTDAT (https://www.inptdat.de/). The idea is to re-use the collected experiment metadata (created with Adamant and stored in the eLabFTW ELN) and bundle it with the dataset for publication *. *Note that the back-end can be easily adapted to use the existing APIs of the mentioned repository platforms to publish metadata, once it is available in a standardized format.
